# Effects of Pre-Sleep Whey vs. Plant-Based Protein Consumption on Muscle Recovery Following Damaging Morning Exercise

**DOI:** 10.3390/nu12072049

**Published:** 2020-07-10

**Authors:** Patrick G. Saracino, Hannah E. Saylor, Brett R. Hanna, Robert C. Hickner, Jeong-Su Kim, Michael J. Ormsbee

**Affiliations:** 1 Department of Nutrition, Food and Exercise Sciences, Institute of Sports Sciences and Medicine, Florida State University, Tallahassee, FL 32306, USA; pgs16@fsu.edu (P.G.S.); saylor16@live.unc.edu (H.E.S.); brh17b@my.fsu.edu (B.R.H.); rhickner@fsu.edu (R.C.H.); jkim6@fsu.edu (J.-S.K.); 2 Discipline of Biokinetics, Exercise and Leisure Sciences, University of KwaZulu-Natal, Durban 4041, South Africa

**Keywords:** dietary protein, plant-based protein, muscle damage, muscle recovery, exercise, strength

## Abstract

Pre-sleep whey protein intake has been shown to improve overnight muscle protein synthesis, muscle size and strength, and muscle recovery. Despite a growing interest in alternative protein sources, such as plant-based protein, there is no evidence regarding the efficacy of plant-based proteins consumed pre-sleep. Therefore, we aimed to compare whey vs. plant-based pre-sleep protein dietary supplementation on muscle recovery in middle-aged men. Twenty-seven recreationally active, middle-aged men performed 5 sets of 15 repetitions of maximal eccentric voluntary contractions (ECC) for the knee extensors (ext) and flexors (flex), respectively, in the morning. Participants consumed 40 g of either whey hydrolysate (WH, *n* = 9), whey isolate (WI, *n* = 6), rice and pea combination (RP, *n* = 6), or placebo (PL, *n* = 6) 30 min pre-sleep on the day of ECC and the following two nights. Catered meals (15% PRO, 55% CHO, 30% Fat) were provided to participants for 5 days to standardize nutrition. Plasma creatine kinase (CK), interleukin-6 (IL-6), and interleukin-10 (IL-10) were measured at pre, immediately post (+0), +4, +6, +24, +48, and +72 h post-ECC. Isometric (ISOM) and isokinetic (ISOK) maximal voluntary contraction force were measured at pre, immediately post (+0), +24, +48, and +72 h post-ECC. Muscle soreness, thigh circumference, and HOMA-IR were measured at pre, +24, +48, and +72 h post-ECC. CK was increased at +4 h post-ECC, remained elevated at all time points compared to baseline (*p* < 0.001), and was significantly greater at +72 h compared to all other time points (*p* < 0.001). IL-6 was increased at +6 h (*p* = 0.002) with no other time differing from baseline. ISOMext was reduced after ECC (*p* = 0.001) and remained reduced until returning to baseline at +72 h. ISOMflex, ISOKext, and ISOKflex were reduced after ECC and remained reduced at +72 h (*p* < 0.001). Muscle soreness increased post-ECC (*p* < 0.001) and did not return to baseline. Thigh circumference (*p* = 0.456) and HOMA-IR (*p* = 0.396) did not change post-ECC. There were no significant differences between groups for any outcome measure. These data suggest that middle-aged men consuming 1.08 ± 0.02 g/kg/day PRO did not recover from damaging eccentric exercise at +72 h and that pre-sleep protein ingestion, regardless of protein source, did not aid in muscle recovery when damaging eccentric exercise was performed in the morning.

## 1. Introduction

Fortunately, there is a growing number of middle-aged and older individuals engaging in exercise and sport [1,2]. However, exercise that is novel or of unaccustomed intensity can elicit muscle damage [3,4] which results in inflammation, muscle soreness, limb swelling, and force loss. These symptoms may persist for several days, compromising subsequent physical activity following a damaging exercise bout. Previous reports have suggested that aging can alter the response to damaging exercise [5,6]. Aging may also be associated with decrements in physical function thereby reducing quality of life [7], though exercise can attenuate these decrements. Thus, elucidating methods to improve muscle recovery from exercise induced muscle damage may be of particular importance to middle-aged people to maintain healthy lifestyles.

Dietary protein supplementation is a widely used nutritional intervention to promote lean mass accrual, strength gains, and help improve recovery. Protein supplementation increases muscle protein synthesis (MPS) and improves net protein balance, improving muscle reconditioning. In fact, a recent meta-analysis determined that protein supplementation had a small to moderate effect on muscle recovery [8]. Research in the past decade has indicated pre-sleep protein consumption as a viable method for increasing overnight MPS, lean mass accrual, and muscle recovery [9,10,11,12,13,14,15] without altering fat metabolism [16,17] or sleep quality [17,18,19].

Groen et al. was the first to show pre-sleep casein protein was effectively digested and absorbed during the overnight period, resulting in increased MPS and net protein balance in older males [9]. Similar results have been reported in young [10] and middle-aged [20] men. Following 12 weeks of resistance exercise (RE), a pre-sleep dairy protein containing beverage (27.5 g casein PRO, 15 g CHO) increased strength and cross sectional area of the quadriceps in recreationally active, young males compared to non-caloric placebo [11]. West et al. provided 25 g of whey protein or CHO following a night-time RE (2000 h) bout to young, resistance trained males [12]. These authors reported improved net protein balance and beneficial effects for protein on MVC (ES: 0.76), Wingate peak power (ES: 0.55), and repetitions to failure (ES: 0.44) 24 h post-exercise. Abbott et al. provided a larger bolus of 40 g pre-sleep casein protein or CHO to trained soccer athletes following night-time soccer matches in a crossover design [13]. Pre-sleep casein protein resulted in a beneficial effect of protein for muscle soreness (large), countermovement jump (small to moderate), and reactive strength index (large) in the 60 h following exercise.

To date, all studies investigating pre-sleep protein have used dairy-based (whey and casein) proteins. However, the use of plant-based proteins has grown in popularity in recent years. The source of protein consumed is an important factor in determining the efficacy of the supplement. Previous research comparing animal-based protein to plant-based protein has shown animal-based protein to elicit greater MPS responses [21,22,23,24,25,26]. It has been suggested that one potential reason for the lower anabolic response of plant-based proteins is a deficiency of one or more essential amino acids [27,28]. When two or more plant-based protein sources are combined this deficiency can be overcome, which is in line with current recommendations to consume multiple plant-based protein sources to provide a complete amino acid profile. Despite the rise in popularity of alternative protein sources, to our knowledge no studies have investigated the efficacy of plant-based protein combinations on muscle recovery or the efficacy of pre-sleep plant-based protein. Therefore, the purpose of the present study was to compare whey protein and plant-based, rice and pea combination protein on muscle recovery in middle-aged men. We hypothesized that whey hydrolysate and whey isolate would improve muscle recovery to a greater extent than would plant-based combination protein, and that all protein sources would outperform placebo.

## 2. Materials and Methods

### 2.1. Participants

Middle-aged (40–64 years), recreationally active (self-reported planned physical activity ≥2 days/week for the past 6 months) healthy men were recruited to participate in this study (*N* = 32). While no exact definition of middle-aged exists, 65 years of age is often considered elderly. Therefore 65 years of age was used as the upper limit of the present study. Participants were excluded if they engaged in eccentric specific exercise of the lower limbs in the past 6 months, chronically took any anti-inflammatory medication, or had a musculoskeletal injury in the past 3 years that would inhibit performance and completion of this study. Additionally, participants were excluded if they had uncontrolled cardiovascular disease (CVD) or metabolic disorders, if they had a BMI ≥ 30 kg/m^2^ and a body fat percentage ≥ 28%, or if they had allergies to dairy products. Lastly, participants were excluded if they smoke or quit smoking in the past 6 months. All participants were informed of the procedures and risks associated with participating and signed an informed consent indicating their understanding. All procedures were approved by the Florida State University Institutional Review Board (Approved December 2017; HSC 2019.26896) prior to testing.

### 2.2. Experimental Protocol

**Figure 1:**
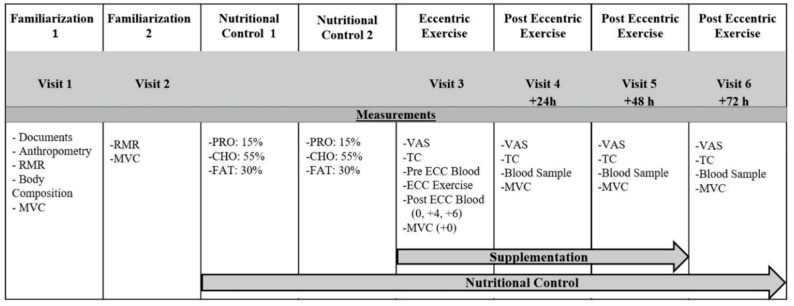
Study Procedures Overview. h = hour; RMR = resting metabolic rate; MVC = maximal voluntary contraction; VAS = visual analogue scale; PRO = protein; CHO = carbohydrate; TC = thigh circumference; ECC = eccentric.

Participants visited the laboratory on 6 occasions. The first 2 visits were for familiarization and included baseline measurements for height, weight, body composition, resting metabolic rate, and isometric (ISOM) and isokinetic (ISOK) maximal voluntary contraction force. On visit 3, participants performed the maximal eccentric exercise bout (ECC). Visits 4 (+24 h), 5 (+48 h), and 6 (+72 h) for post-ECC follow up testing for muscle soreness, thigh circumference, blood sampling, and ISOK and ISOM. For an overview of the study procedures, see Figure 1. Due to the relatively large age range utilized in the present study and the potential impact of both age and body fat percentage on outcome variables, participants were stratified by both age and body fat percentage then semi-randomly (matched by age and body fat percentage) assigned (*N* = 8/group) to either whey hydrolysate (WH), whey protein isolate (WI), rice and pea combination (RP), or a flavor matched, non-caloric placebo (PL) 30 min pre-sleep (40 g) on the ECC day (visit 3) and the two subsequent experimental trial days (visit 4 (+24 h), visit 5 (+48 h)). Water was provided ad libitum during all visits. To increase ecological validity, a 25 g bolus of the assigned supplement was provided immediately following the post-ECC blood sample. A standardized meal (396 kcal, 5 g PRO, 76 g CHO, 8 g fat) was also provided to all participants 2 h following completion of ECC. Protein supplements were weighed, packaged, and labeled by a researcher not otherwise involved in this study. Prior to leaving the laboratory, participants received a supplement package and an opaque bottle containing ~350 mL of water with instruction as to how and when to mix and consume the beverage. Participants were instructed to write the time of consumption on the provided package and bring the empty package to the laboratory in the morning to indicate compliance. The energy content and amino acid profile of the pre-sleep protein drinks can be found in Table S1.

### 2.3. Resting Metabolic Rate

Participants arrived at the laboratory in the morning of visits 1 and 2 in the overnight fasted state. Participants were asked to refrain from caffeine consumption for 12 h and physical activity and alcohol consumption for 24 h prior to all metabolic measurements. Upon arrival, height and weight measurements were recorded. Testing was performed in a dark, climate-controlled room (20–23 °C). Resting metabolic rate (RMR) was measured with computerized open-circuit indirect calorimetry ParvoMedics TrueOne 2400 metabolic cart, Sandy, UT, USA) using a fitted hood. Gas exchange was measured continuously for 30 min and measurements recorded in the final 20 min were used for data analysis. Prior to testing, calibrations were performed with a flow meter using a 3-L syringe and with gas analyzers using verified gases of known concentrations according to manufacturer guidelines.

### 2.4. Maximal Voluntary Contraction

Testing was performed on the Biodex isokinetic dynamometer (Biodex Medical Systems, Shirley, NY, USA) on all visits. The participant was positioned so that the backrest was firmly against the back and the hips flexed at 90°. Hip, thigh, and chest straps were tightened so the participant was firmly secured into place. The lever arm was aligned visually at the axis of rotation of the knee joint at the lateral epicondyle. The lever arm was extended so that the inferior rim of the ankle pad was contacting the tibia just above the malleoli of the ankle. The positioning was recorded for each participant and utilized for all subsequent testing. The range of motion for extension and flexion was set. The leg was extended, locked into place, and the weight of the limb was recorded.

Participants performed 5 isokinetic (ISOK) maximal voluntary contractions (MVC) of concentric extension and flexion, respectively, at 60°/s. Two minutes of rest was provided then the participants limb was moved to 60°. Three total repetitions were performed for isometric MVC (ISOM) for each extension and flexion. Repetitions were performed as 5 s MVC extension followed by 5 s of rest then 5 s of MVC flexion until all 3 repetitions were completed. The greatest peak torque for ISOM and ISOK, respectively, was used in data analysis. Baseline measurements during visits 1 and 2 utilized both limbs to determine maximal concentric torque of both limbs in order to assess effort during the eccentric exercise on visit 3. Only the dominant leg was tested during post-ECC time points.

### 2.5. Muscle Soreness

Prior to blood sampling, participants were seated on a bench and asked to perform a full unweighted knee extension and knee flexion of their dominant limb. They were asked to rate their perceived soreness in the muscles during the entire movement by making a vertical line on an anchored 100 mm visual analogue scale at pre, +24, +48, and +72 h post-ECC. The scale was labeled with “no soreness” at 0 mm and “extreme soreness” at 100 mm.

### 2.6. Thigh Circumference

A Gullick tape measure was used to quantify thigh circumference on the dominant leg of each participant. Circumference was measured while placing the foot on a bench with the knee bent at a 90° angle. An ink mark was made on the skin at the midpoint between the proximal border of the patella and the intersection of the inguinal crease and anterior midline of the thigh to ensure measurements were taken in the same location during each trial.

### 2.7. Blood Sampling

Blood was collected from an antecubital vein (10 mL) at pre, post (+0), +4, +6, +24, +48, and +72 h post-ECC into vacutainer tubes lined with EDTA (Becton, Dickinson and Company, New Jersey, USA). Blood was centrifuged (Thermo Scientific, Waltham, MA) for 15 min at 3500 rpm at 4 °C. Plasma aliquots (300–500 µL) were transferred into microtubes and stored at −80 °C for later analysis. IL-10, insulin (Cat. Nos. DINS00, SS100C; R&D Systems, Minneapolis, MN, USA), and CK (Cat. No. MAK116, Sigma Aldrich, St. Louis, MO, USA) concentrations were determined in duplicate by immunoassay according to manufacturer instructions. Glucose and IL-6 concentrations were measured using a Beckman Coulter DxC600i. Sensitivities were 0.17 pg/mL, 2.15 pmol/L, 30 U/L, 5 mg/dL, and 0.5 pg/mL, respectively. Glucose and insulin concentrations were used to calculate HOMA-IR.

### 2.8. Muscle Damage Protocol

On visit 3, participants performed 5 sets of 15 repetitions of maximal voluntary eccentric contractions of the knee extensors and knee flexors, respectively, for each leg at 60°/s on an isokinetic dynamometer. Two minutes of rest was given between sets and 5 min between each leg. Visual feedback was provided during the entirety of the eccentric exercise bout and verbal encouragement was provided by the researchers to promote maximal effort.

### 2.9. Dietary and Exercise Control

Caloric needs were determined from RMR data and adjusted with an activity factor of 1.375. Participants were provided a standardized diet of 15% PRO, 55% CHO, and 30% fat (Vale Food Company, Tallahassee, FL, USA) 2 days prior to and during all eccentric exercise and experimental days for a total of 5 days. All meals were designed by a registered dietitian. Participants were asked to refrain from exercise outside of the study for 24 h prior to any testing and on any days where meals were provided.

### 2.10. Statistics

A priori power analysis (G*Power 3.1; Dusseldorf, Germany) for isometric peak torque was performed based on a similar study investigating pre-sleep protein on muscle recovery. The authors reported a Cohen’s *d* effect size of 0.28 for isometric peak torque at 10 h post-exercise [12]. Using an α level of 0.05 and β of 0.8 with 4 groups and 5 timepoints, it was determined that total sample size to detect differences in the mixed repeated measures ANOVA was 28 participants i.e., 7 participants per group. However, in order to ensure sufficient power of the present study and to account for attrition, we intended to recruit 8 individuals per group. A one-way ANOVA was utilized to determine if differences between groups existed at baseline for all participant characteristics, standardized nutrition, and outcome variables. Mixed repeated measures ANOVA was used to determine differences for all outcome variables. All measures contained 4 groups (WH, WI, RP, PL). There were 5 timepoints (pre, post, +24, +48, +72) for muscle function, 7 timepoints (pre, post, +4, +6, +24, +48, +72) for blood variables, and 4 timepoints (pre, +24, +48, +72) for muscle soreness and thigh circumference. As a secondary analysis, whey protein groups were condensed and compared to plant-based protein and placebo. For all analyses using more than 2 groups, Bonferroni post hoc analysis was performed to determine between group differences for significant group x time interactions, if they existed. For secondary analyses, post hoc pairwise comparisons using Bonferroni corrections were utilized to further investigate significant group x time interactions if comparing only 2 groups. If sphericity was violated, Greenhouse-Geisser corrections were used. All group x time interactions and main effects are reported as (F_dftreatment, dferror_) = F statistic, *p*-value, partial eta squared (η_p_^2^). Significant main effects were further investigated using Bonferroni pairwise comparisons. For ηp^2^, effect size values were qualified as follows: small = 0–0.02, medium = 0.02–0.13, large = 0.13–0.26. Statistical analysis was completed using SPSS (IBM SPSS Statistics for Windows, version 25; IBM Corp, Chicago, IL, USA). Retrospective power analysis was performed using SPSS. Data are reported as mean ± standard error of the mean (SEM). Significance was set at *p* < 0.05.

## 3. Results

### 3.1. Participant Characteristics

Thirty-two middle-aged recreationally active men were recruited from the Tallahassee, Florida community and volunteered to participate in the study. One participant subsequently indicated they have sickle cell trait and was dropped from the study due to diagnosis of rhabdomyolysis prior to completion of the study. Those data were removed from analysis. A second participant was diagnosed with rhabdomyolysis after completion of the study. Those data were included in final analyses. Of the two participants diagnosed with rhabdomyolysis, one was in RP and the other in PL. Both individuals recovered fully. While 32 participants were recruited, the study was terminated due to safety concerns from the eccentric exercise protocol. Thus, 27 individuals completed the entire protocol and were included in final analyses. There were no significant differences at baseline between groups for age, height, weight, lean mass, or body fat % (Table 1).

### 3.2. Nutrient Intake

Mean energy intake for all participants during the 5 days of standardized nutrition was set at 15%, 55%, and 30% of total daily calories for PRO, CHO, and fat, respectively. Mean energy intake was 2351 ± 60 kcals (PRO: 88 ± 2 g (1.08 ± 0.02 g/kg), CHO: 323 ± 8 g, FAT: 78 ± 2 g). There were no differences between treatments for calories, PRO, PRO g/kg, CHO, CHO g/kg, fat, or fat g/kg intake (Table 2). RP consumed a significantly greater percentage of protein from plant sources and a significantly lower percentage from animal sources compared to all other groups (*p* < 0.001).

### 3.3. Muscle Function

#### 3.3.1. ISOMext

**Figure 2:**
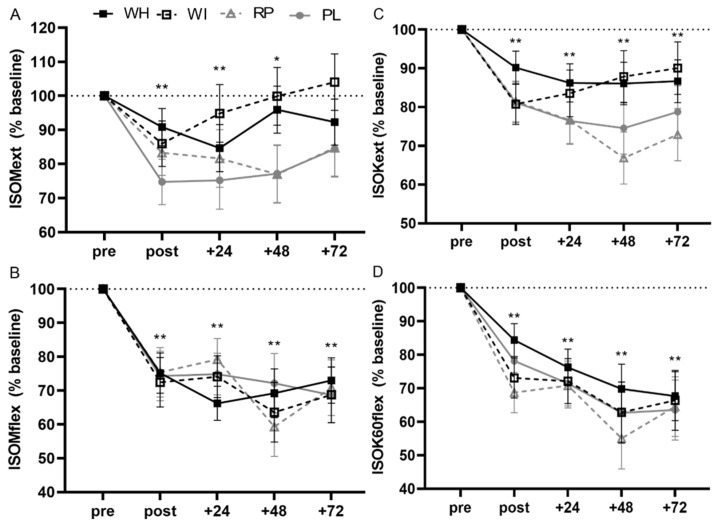
Isometric extension (**A**), Isometric flexion (**B**), Isokinetic extension 60°/s (**C**), Isokinetic flexion 60°/s (**D**). Raw data was analyzed for statistical purposes and represented as percent of baseline values; ** indicates significant main time effect from baseline (*p* ≤ 0.01); * indicates significant main time effect from baseline (*p* ≤ 0.05).

One-way ANOVA indicated that there were no significant differences between groups for baseline power (*p* = 0.867). There were no significant group x time interactions (F_8.191, 62.801_ = 1.291, *p* = 0.263, η_p_^2^ = 0.144) or main group effects (F_3, 23_ = 0.227, *p* = 0.876, η_p_^2^ = 0.029). There was an observed power of 0.55 for the interaction. There were significant main time effects following the eccentric exercise bout (F_2.730, 62.801_ = 10.366, *p* < 0.001, η_p_^2^ = 0.311). Peak torque was reduced immediately post-ECC and remained reduced compared to baseline until returning to pre-ECC values at +72 h (Figure 2, Table S2). There were no significant group x time interactions (F_5.615, 67.378_ = 1.517, *p* = 0.190, η_p_^2^ = 0.112) or main group effects (F_2, 24_ = 0.326, *p* = 0.725, η_p_^2^ = 0.026) when whey protein groups were combined and compared to the plant-based group and placebo. Peak torque was reduced post-ECC (*p* < 0.01) and trended to remain reduced at +72 (*p* = 0.082).

#### 3.3.2. ISOMflex

One-way ANOVA indicated that there were no significant differences between groups for baseline power (*p* = 0.675). There were no significant group x time interactions (F_8.524, 65.347_ = 1.031, *p* = 0.424, η_p_^2^ = 0.118) or main group effects (F_3, 23_ = 0.317, *p* = 0.819, η_p_^2^ = 0.040). There was an observed power of 0.453. There were significant main time effects following the eccentric exercise bout (F_2.841, 65.347_ = 34.134, *p* < 0.001, η_p_^2^ = 0.597). Peak torque was reduced at all time points following the eccentric exercise bout and did not return to pre-ECC values at +72 h (Figure 2, Table S2). There were no significant group x time interactions (F_8, 96_ = 1.167, *p* = 0.327, η_p_^2^ = 0.089) or main group effects (F_2, 24_ = 0.438, *p* = 0.651, η_p_^2^ = 0.035) when whey protein groups were combined and compared to the plant-based group and placebo. Peak torque was still reduced compared to baseline at +72 (*p* < 0.001).

#### 3.3.3. ISOK60ext

One-way ANOVA indicated that there were no significant differences between groups for baseline power (*p* = 0.632). There were no significant group x time interactions (F_7.510, 57.573_ = 1.593, *p* = 0.151, η_p_^2^ = 0.172) or main group effects (F_3, 23_ = 0.784, *p* = 0.515, η_p_^2^ = 0.093). There was an observed power of 0.629 for the interaction. There were significant main time effects following the eccentric exercise bout (F_2.503, 57.573_ = 19.258, *p* < 0.001, η_p_^2^ = 0.456). Peak torque was reduced at all time points following the eccentric exercise bout and did not return to pre-ECC values at +72 h (Figure 2, Table S2). There were no significant group x time interactions (F_5.132, 61.582_ = 1.948, *p* = 0.097, η_p_^2^ = 0.140) or main group effects (F_2, 24_ = 0.775, *p* = 0.472, η_p_^2^ = 0.061) when whey protein groups were combined and compared to the plant-based group and placebo. Peak torque was still reduced at +72 compared to baseline (*p* < 0.001).

#### 3.3.4. ISOK60flex

One-way ANOVA indicated that there were no significant differences between groups for baseline power (*p* = 0.842). There were no significant group x time interactions (F_6.417, 49.196_ = 0.727, *p* = 0.639, η_p_^2^ = 0.087) or main group effects (F_3, 23_ = 0.827, *p* = 0.827, η_p_^2^ = 0.037). There was an observed power of 0.268 for the interaction. There were significant main time effects following the eccentric exercise bout (F _2.139, 49.196_ = 39.055, *p* < 0.001, η_p_^2^ = 0.629). Peak torque was reduced at all time points following the eccentric exercise bout and did not return to pre-ECC values at +72 h (Figure 2, Table S2). There were no significant group x time interactions (F_4.361, 52.330_ = 0.922, *p* = 0.465, η_p_^2^ = 0.071) or main group effects (F_2, 24_ = 0.385, *p* = 0.685, η_p_^2^ = 0.031) when whey protein groups were combined and compared to the plant-based group and placebo. Peak torque was still reduced at +72 compared to baseline (*p* < 0.001).

### 3.4. Blood Markers

#### 3.4.1. Creatine Kinase (CK)

**Figure 3:**
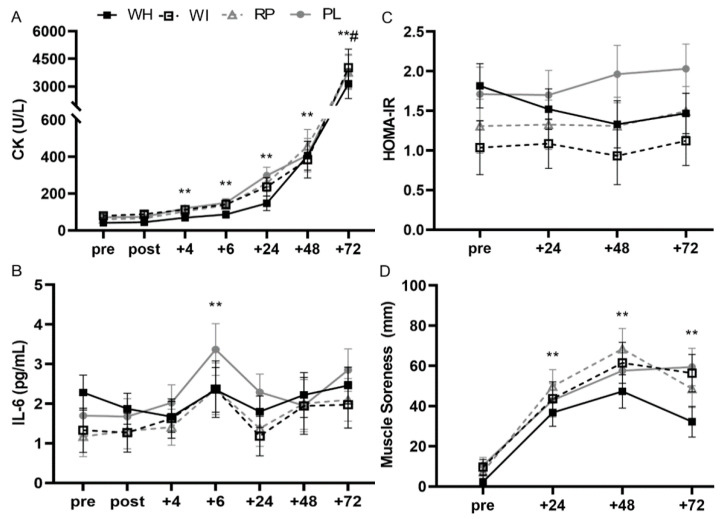
Plasma creatine kinase concentration (U/L; **A**), plasma interleukin 6 concentration (pg/mL; **B**), homeostatic model of assessment insulin resistance (HOMA-IR; **C**) and muscle soreness rating (mm; **D**). ** indicates significant main time effect from baseline (*p* ≤ 0.01); * indicates significant main time effect from baseline (*p* ≤ 0.05); # indicates significantly different from all other time points (*p* ≤ 0.01).

One-way ANOVA indicated that there were no significant differences between groups at baseline (*p* = 0.147). There were no significant group x time interactions (F_3.016, 21.113_ = 0.184, *p* = 0.907, η_p_^2^ = 0.026, *n* = 25) or main effects of group (F_3, 21_ = 0.275, *p* = 0.843, η_p_^2^ = 0.038. There was an observed power of 0.079 for the interaction. There was a main effect of time (F_1.005, 21.113_ = 62.385, *p* < 0.001, η_p_^2^ = 0.748). CK increased at +4 h (*p* < 0.001) and remained elevated at all time points compared to baseline, with a significant increase from all other time points at +72 h (*p* < 0.001; Figure 3). There were no significant group x time interactions (F_2.013, 22.141_ = 0.057, *p* = 0.945, η_p_^2^ = 0.005) or main group effects (F_2, 22_ = 0.131, *p* = 0.878, η_p_^2^ = 0.012) when whey protein groups were combined and compared to the plant-based group and placebo. CK was elevated at +4 h (*p* < 0.001) and remained elevated at all time points compared to baseline, increasing at all time points until reaching a peak at +72 h. Intra and Inter assay CVs for CK were 3.25% and 2.92%, respectively.

#### 3.4.2. IL-6

One-way ANOVA indicated that there were no significant differences between groups at baseline (*p* = 0.535). There were no significant group x time interactions (F_9.961, 69.728_ = 0.0.854, *p* = 0.579, η_p_^2^ = 0.109, *n* = 25) or main effects of group (F_3, 21_ = 0.422, *p* = 0.739, η_p_^2^ = 0.057). There was an observed power of 0.408 for the interaction. There was a main effect of time (F_3.320, 69, 728_ = 7.633, *p* < 0.001, η_p_^2^ = 0.267). IL-6 concentrations were significantly greater at +6 h compared to baseline (*p* = 0.002). IL-6 returned to baseline at +24 h and trended to increase again at +72 h compared to baseline (*p* = 0.089; Figure 3). There were no significant group x time interactions (F_6.842, 75.262_ = 0.966, *p* = 0.461, η_p_^2^ = 0.081) or main group effects (F_2, 22_ = 0.426, *p* = 0.658, η_p_^2^ = 0.037) when whey protein groups were combined and compared to the plant-based group and placebo. IL-6 concentrations increased compared to baseline at +6 h (*p* = 0.002), returned to baseline, then increased again at +72 h (*p* = 0.048). Interestingly, when whey was compared to PL only, there was a trend for a significant group x time interaction (F_3.976, 67.597_ = 2.276, *p* = 0.070, η_p_^2^ = 0.118) but no significant group x time interaction when plant was compared to PL only (F_2.255, 22.549_ = 0.501, *p* = 0.635, η_p_^2^ = 0.048). Upon visual observation, WH was compared to PL only and there was a significant time x treatment interaction (F_6, 72_ = 2.228, *p* = 0.050, n_p_^2^ = 0.157). However, further analysis of this interaction revealed there were no differences between WH and PL at any time point. Samples were not analyzed in duplicate; thus, no CV was calculated.

#### 3.4.3. IL-10

All measured concentrations were below the valid assay range (0.8 pg/mL). Thus, these data were not analyzed.

#### 3.4.4. HOMA-IR

One-way ANOVA indicated that there were no significant differences between groups at baseline (*p* = 0.306; *n* = 27). There were no significant group x time interactions (F_7.037, 53.950_ = 1.559, *p* = 0.167, η_p_^2^ = 0.169), main effects of group (F_3, 23_ = 1.183, *p* = 0.338, η_p_^2^ = 0.134), or main effects of time (F_2.346, 53.950_ = 1.005, *p* = 0.383, η_p_^2^ = 0.042; Figure 3). There was an observed power of 0.595 for the interaction. There were no significant group x time interactions (F_4.614, 55.365_ = 1.689, *p* = 0.157, η_p_^2^ = 0.123), main effects of group (F_2, 24_ = 1.014, *p* = 0.378, η_p_^2^ = 0.078), or time (*p* = 0.374) when whey protein groups were combined and compared to the plant-based group and placebo. Interestingly, when whey was compared to PL only, there was a trend for a significant group x time interaction (F_2.265, 43.042_ = 3.051, *p* = 0.052, η_p_^2^ = 0.138) but no significant group x time interaction when plant was compared to PL only (F_3, 30_ = 0.503, *p* = 0.683, η_p_^2^ = 0.048). Additionally, upon visual observation, WH was compared to PL only and there was a significant group x time interaction (F_3, 39_ = 3.410, *p* = 0.027, η_p_^2^ = 0.208). However, further analysis of this interaction revealed there were no differences between WH and PL at any time point. Intra and Inter assay CVs for insulin were 2.81% and 0.98%, respectively. Glucose samples were not analyzed in duplicate; thus, no CV was calculated.

### 3.5. Muscle Soreness

One-way ANOVA indicated that there were no significant differences between groups at baseline (*p* = 0.330). There were no significant group x time interactions (F_9, 69_ = 0.780, *p* = 0.636, η_p_^2^ = 0.092) or main group effects (F_3, 23_ = 1.669, *p* = 0.201, η_p_^2^ = 0.179). There was an observed power of 0.353 for the interaction. There were significant main time effects following the eccentric exercise bout (F_3, 69_ = 49.690, *p* < 0.001, η_p_^2^ = 0.684). Muscle soreness was increased following the eccentric exercise bout and did not return to pre-ECC values at +72 h (Figure 3). There were no significant group x time interactions (F_6.293, 71.677_ = 0.966, *p* = 0.461, η_p_^2^ = 0.081) or main group effects (F_2, 22_ = 3.656, *p* = 0.658, η_p_^2^ = 0.037) when whey protein groups were combined and compared to the plant-based group and placebo.

### 3.6. Thigh Circumference

One-way ANOVA indicated that there were no significant differences between groups at baseline (*p* = 0.261). There were no group x time interactions (F_5.463, 41.884_ = 1.027, *p* = 0.418, η_p_^2^ = 0.118), main group effects (F_3, 23_ = 1.452, *p* = 0.254, η_p_^2^ = 0.159), or main time effects (F_1.821, 41.884_ = 0.880, *p* = 0.413, η_p_^2^ = 0.037). There was an observed power of 0.343 for the interaction. There were no significant group x time interactions (F_3.716, 44.588_ = 1.138, *p* = 0.349, η_p_^2^ = 0.087) or main group effects (F_2, 24_ = 2.002, *p* = 0.157, η_p_^2^ = 0.143) when whey protein groups were combined and compared to the plant-based group and placebo.

## 4. Discussion

Previous studies report increased MPS following ingestion of 40 g of dairy-based protein 30 min pre-sleep, which results in improved net protein balance and muscle reconditioning in the overnight window [14,29]. While MPS can serve as a proxy for muscle reconditioning, it may not be an appropriate estimate of chronic muscular adaptation [25]. Importantly, recent investigations have reported pre-sleep whey [12] and casein [13] protein improves muscle recovery following exercise induced muscle damage and improvements in overnight net protein balance coincide with improved functional recovery [12]. As all pre-sleep protein supplement studies to date have used dairy-based protein sources, the present study was designed to compare the effects of whey protein and plant-based combination protein on muscle recovery in middle-aged men following damaging eccentric exercise. The primary findings of the present study were that whey and plant-based protein consumed pre-sleep failed to consistently improve muscle function during the 72 h recovery period and whey and plant-based protein consumed pre-sleep failed to reduce blood markers of muscle damage and inflammation or muscle recovery. In contrast to our hypotheses, pre-sleep whey proteins were not more effective at improving muscle function, markers of inflammation and muscle damage, muscle soreness, or metabolism compared to plant-based protein and pre-sleep protein, regardless of source, was not more effective than placebo.

As expected, the eccentric protocol utilized in the present study resulted in reduced peak torque in all muscle function variables, indicating damage. Force reduction following intensive exercise has been well reported [12,13,30,31,32,33,34]. Pre-sleep protein supplementation has been suggested to attenuate reductions in force production [12,13]; however, the present data do not support benefits of pre-sleep protein on attenuating reductions in isometric or isokinetic peak torque. It is possible that differences in results are attributed to the use of different outcome measures as Abbott et al. [13] utilized reactive strength index and countermovement jump as markers of muscle function while in the present study outcome measures were peak torque of the knee extensors and flexors. West et al. [12] utilized peak isometric force of the knee extensors and yet there were still discrepant findings despite similar measures to the present study, making this an implausible explanation. The discrepancy in age of participants between studies could explain differences in findings, as both studies reporting benefits of pre-sleep protein on muscle recovery examined effects in younger populations. Aging has, indeed, been shown to alter muscle damage [5,6] and the response to protein feeding [35,36]. Thus, studies examining the muscle recovery effects of pre-sleep protein in younger individuals may not be translatable to older populations. Holwerda et al. [37] provided a pre-sleep whey protein treatment containing 21 g of leucine enriched protein (21 g PRO, 9 g CHO, 3 g fat; 3 g total leucine) compared to an energy matched carbohydrate control (0 g PRO, 25 g CHO, 6 g fat) during 12 weeks of whole-body resistance training in 41 older (70 ± 1 y) males. Resistance training was performed 3 times per week between 0800 and 1100 h. While lean mass, quadriceps cross sectional area, type II fiber cross sectional area, and 1RM strength increased with training, there was no benefit of pre-sleep protein supplementation compared to control. It should be noted that previous literature has shown neither 20 g nor 30 g casein protein with or without additional leucine to be sufficient to increase MPS during the overnight period, suggesting a minimum dose of 40 g is required for this time frame [19,38]. Thus, it is possible that the 21 g of leucine enriched protein was insufficient to induce benefit. Participants ingested a larger bolus of pre-sleep protein in the present study and still did not receive a measurable benefit compared to placebo. Therefore, the present study agrees with the findings of Holwerda et al. [37] that pre-sleep protein does not seem to have a benefit for older populations when exercise is performed in the morning hours.

It has been suggested that total daily protein consumption rather than protein timing may be the primary contributor to effects of protein on both muscle size and recovery [39,40]. Following 12 weeks of resistance training, muscle strength, quadriceps cross sectional area, and type II muscle fiber size increased to a greater extent compared to placebo when a protein supplement (27.5 g casein, 15 g carbohydrate) was consumed before sleep [11]; however, the protein group consumed 1.9 ± 0.1 g/kg of protein compared to 1.3 ± 0.1 g/kg in the control group. It is therefore difficult to attribute the observed beneficial effects solely to the pre-sleep protein supplementation. The present study employed rigorous nutritional control by providing participants catered meals managed by a registered dietitian. These meals were designed to provide 15% of their total daily caloric intake from protein, inclusive of the pre-sleep supplement. This led to a mean intake of 1.08 g/kg, which is above the RDA (0.8 g/kg). While the 15% of total daily calories from protein (1.08 ± 0.02 g/kg mean protein intake) is in range of what adults typically consume in the United States [41] and is suggested as the RDA, it is below the suggested optimal range. It is now known that the optimal daily protein intake is closer to 1.2–1.6 g/kg [36] or 1.4–2.0 g/kg [42]. Therefore, it is possible that the lack of benefit from pre-sleep protein in the present study can be attributed to the daily protein intake of our participants. In the current study, nearly all muscle function outcomes were still reduced at 72 h post-ECC, indicating muscle damage and, therefore, lack of recovery. Our data indicate that our participants did not recover fully within 72 h. It is possible that we missed the window to see recovery changes in this population. It could also be that higher daily protein intakes than were provided are required to elicit muscle recovery in middle aged men. Although the lower than optimal protein provided could be looked at as a limitation, the authors believe it adds to the practicality of our findings as the protein provided was in line with reported intakes for individuals of this age [41,43]. These data suggest that the average middle-aged male consumes insufficient protein to elicit recovery benefits for up to 72 h following intense exercise. Future work should be completed in this population to determine optimal protein intake ranges for muscle recovery. While the consumption of lower than optimal daily protein is a viable argument for the lack of benefit of pre-sleep protein observed in the present study, recent work is indicating that the timing of exercise in relation to protein consumption may be an explanation for our findings.

Importantly, the time of exercise training appears to be a critical factor for observing an efficacious response on recovery from pre-sleep protein. Work examining pre-sleep protein utilizing night-time exercise, whereby the protein supplement is consumed in close proximity to the training stimulus, has shown benefit [10,11,12,13,15]. West et al. [12] examined the effects of 25 g of whey consumed immediately after an evening (2000 h) exercise session and again the following morning compared to energy matched carbohydrate supplement. Protein improved 24 h protein balance compared to placebo. Further, protein showed a moderate beneficial effect for MVC, repetitions to failure, and Wingate peak power at 24 h. Abbott et al. [13] provided 40 g of casein protein or carbohydrate control pre-sleep, in a crossover design, to young male soccer players at night following a soccer competition (1900 h). In the night-time protein group, countermovement jump and reactive strength index decrements were attenuated and muscle soreness was reduced compared to isocaloric carbohydrate during a 60 h recovery period. However, in the present study, the eccentric exercise session was performed in the morning, approximately 10+ h prior to consumption of the pre-sleep protein which differs from West et al. and Abbott et al. Upon further review, data is mounting to support that utilizing a morning exercise bout rather than night-time exercise reduces or eliminates the effectiveness of pre-sleep protein on muscle recovery [34,37,44]. In support of this claim, Apweiler et al. [34] utilized 100 drop jumps performed in the morning (between 0730 and 0900 h) to induce muscle damage in young males and females. A bolus of 40 g casein protein or carbohydrate control was consumed pre-sleep. While reductions in muscular performance following the bout of drop jumps were noted, indicating muscular damage, no benefits were reported for protein consumption in the 48 h following exercise. This finding was despite the fact the protein group consumed greater total protein than the control group (2.12 ± 0.51 vs. 1.60 ± 0.59 g/kg, respectively). Similarly, Larsen et al. [44] provided 0.5 g/kg of either pre-sleep whey protein isolate or carbohydrate to trained male runners that performed 11 endurance training sessions in the morning and afternoon over 1 week. Due to exercise being performed in the morning and afternoon, pre-sleep protein supplementation was provided hours after the training stimulus. Nutritional control was provided by the researchers and protein intake in both groups was set at 1.8 g/kg. No benefit was observed for time trial performance, training volume, or blood biomarkers (CK, LDH, Mb) between groups. The present study agrees with these findings to show no benefit of pre-sleep protein when protein is consumed hours away from the training stimulus (i.e., when exercise is performed in the morning but protein is consumed pre-sleep). The exact time between training and pre-sleep protein where protein no longer shows a positive effect has not been investigated. As pre-sleep protein studies have utilized younger [34,44] and older [37] male populations, we can now extend this concept to muscle recovery in middle-aged men. The present study also expands upon the current literature by indicating pre-sleep protein, regardless of source, does not have benefit on muscle recovery when exercise is performed in the morning. Since prior evening exercise has been shown to augment amino acid incorporation into myofibrillar protein with pre-sleep protein consumption [15], it can be speculated that the lack of benefit with morning exercise can be, in part, attributed to reduced amino acid incorporation into skeletal muscle, though, this has not been examined. Some athletic events and training sessions take place at midday, thus future research should investigate these types of training scenarios with pre-sleep protein supplementation. Additionally, both whey and casein proteins have been utilized in the pre-sleep protein literature. We have previously reported no differences between pre-sleep whey and casein ingestion [45,46] yet no direct comparison has been made between the two dairy protein sources for MPS or muscle recovery when consumed pre-sleep. Future work should investigate the efficacy of pre-sleep whey compared to casein protein on MPS and muscle recovery.

The major limitation of the present study is the small sample size. While the intent was to recruit 8 individuals per group, this study was discontinued early to preserve the health and safety of participants. To that end, not all participants completed the protocol due to 2 cases of rhabdomyolysis leaving the present study underpowered at 55% for the primary outcome of ISOMext rather than 80% as designed, making interpretation of the findings difficult. Further, it appears that the protocol used in the present study cannot be deemed safe for this population and caution should be taken when designing muscle damaging protocols, particularly in middle-aged men. While a similar ECC protocol has been cited as being effective at inducing muscle damage in recreationally trained young men [47], it may lead to excessive damage and pursuant rhabdomyolysis in recreationally active middle-aged men, as was observed in the present study. Along with intense exercise, various other factors including hot ambient temperatures, dehydration, and alcohol use have been suggested to induce rhabdomyolysis and related kidney dysfunction [48,49]. Therefore, it is possible that factors beyond the novel and accustomed nature of the eccentric exercise bout contributed to the diagnoses of rhabdomyolysis in the present study. It should be noted that one of the participants who suffered from rhabdomyolysis during this protocol was found to have sickle cell trait, indicating that the protocol should not be used in individuals with sickle cell anemia or sickle cell trait. Though water was provided ad libitum during all visits, blood variables were not adjusted to account for plasma shifts. It is possible that plasma shifts could have altered the measured concentrations. Further, only male participants between 40 and 64 years of age were included in the present study, limiting the generalizability of findings to middle-aged men only. Future work should compare the effectiveness of whey vs. plant-based protein in women. Though there were no significant differences between groups for age, it is possible that the wide age range utilized in the present study altered our findings. Future work should investigate smaller age groups and comparisons of younger vs. older individuals with pre-sleep protein supplementation. Additionally, only the protein supplement was plant or animal based, and not the standard diet. Thus, the standardized diet contained animal-based protein and was the same for all groups. Therefore, individuals in the RP group consumed animal-based proteins as part of their standardized diet which may have altered our findings. However, this was required to truly blind the study as it was designed but it leaves the possibility that the animal-based whole food protein provided in the standardized diet was sufficient to overpower any differences due to animal-based vs. plant-based pre-sleep protein supplementation. Future studies should investigate standardized nutrition meals void of animal-based protein as to determine the effects of consuming a diet void of animal-based protein (i.e., vegan) compared to a diet with animal-based protein.

## 5. Conclusions

Pre-sleep protein, regardless of source, did not aid in muscle recovery when damaging eccentric exercise was performed in the morning. Middle-aged men consuming daily protein intakes in line with previously reported averages (~90g/day) for this age group [41,43] and above the current recommendation for daily protein intake, were not recovered 72 h following damaging eccentric exercise. Intakes of greater than 1.08 g/kg are likely required to promote recovery within a middle-aged population, therefore, active middle-aged men should likely consume more protein especially if performing exercise bouts which are novel or of unaccustomed intensity. The results from the present study suggest active, middle-aged men should first prioritize consuming adequate per day intakes of protein (1.2–1.6 g/kg [36] or 1.4–2.0 g/kg [42]) and to consume a protein meal in close proximity with their exercise sessions to promote muscle recovery.

## Figures and Tables

**Table 1:** Participant Characteristics.

	Whey Hydrolysate	Whey Isolate	Rice/Pea	Placebo	Total	*p*-Value
*N*	9	6	6	6	27	
Age, yr	57 ± 2	53 ± 3	56 ± 2	52 ± 4	55 ± 1	0.655
Height, cm	179 ± 3	180 ± 3	179 ± 2	180 ± 3	179 ± 1	0.965
Weight, kg	86.1 ± 4.3	83.9 ± 4.4	79.1 ± 4.9	78.7 ± 5.5	82.4 ± 2.3	0.613
Lean Mass, kg	61.7 ± 1.9	60.7 ± 2.5	57.2 ± 3.2	55.9 ± 3.2	59.2 ± 1.3	0.346
Body Fat, %	24 ± 2	23 ± 2	23 ± 1	24 ± 2	23 ± 1	0.967

Data are presented as mean ± SE.

**Table 2:** Standardized Nutrition.

	Whey Hydrolysate	Whey Isolate	Rice/Pea	Placebo	Total
Calories, kcal	2405 ± 113	2439 ± 136	2293 ± 102	2240 ± 135	2351 ± 60
CHO, g	331 ± 16	335 ± 19	315 ± 14	308 ± 19	323 ± 8
CHO, g/kg	3.86 ± 0.14	4.02 ± 0.21	4.03 ± 0.22	3.96 ± 0.21	3.96 ± 0.09
Fat, g	80 ± 4	81 ± 5	76 ± 3	75 ± 5	78 ± 2
Fat, g/kg	0.94 ± 0.03	0.97 ± 0.05	0.98 ± 0.05	0.96 ± 0.05	0.96 ± 0.02
PRO, g	90 ± 4	91 ± 5	86 ± 4	84 ± 5	88 ± 2
PRO, g/kg	1.05 ± 0.04	1.10 ± 0.06	1.10 ± 0.06	1.08 ± 0.06	1.08 ± 0.02
Plant PRO, %	26.1 ± 2.0	28.1 ± 4.9	65.0 ± 2.6 **	31.6 ± 2.0	36.7 ± 3.4
Animal, PRO, %	74.0 ± 2.0	71.9 ± 4.9	35.1 ± 2.6 **	68.4 ± 2.0	63.3 ± 3.4

Data are presented as mean ± SE; % = percent of protein by source; Macronutrients were set to 15% PRO, 55% CHO, 30% fat total daily calories; ** One-way ANOVA indicates significantly different from all other groups (*p* ≤ 0.001).

## Supplementary Materials

The following are available online at https://www.mdpi.com/2072-6643/12/7/2049/s1, Table S1: Pre-Sleep Protein Supplement Composition, Table S2: Muscle Function Raw Data.
