# Supplementary material for: Effects of Pre-Sleep Whey vs. Plant-Based Protein Consumption on Muscle Recovery Following Damaging Morning Exercise
Source: Nutrients. 2020 Jul 10;12(7):2049. doi: 10.3390/nu12072049 (PMC7400837; doi:10.3390/nu12072049)
Supplement: Supplementary file 1 [file nutrients-12-02049-s001.pdf]

Supplemental Table S1. Pre-Sleep Protein Supplement Composition

	Whey Hydrolysate	Whey Isolate	Rice/Pea
**Calories (kcal)**	183.6	185.5	205.0
**Protein (g)**	40.5	40.3	40.4
**Alanine (g)**	2.0	2.0	2.0
**Arginine (g)**	1.1	1.1	3.5
**Aspartic Acid(g)**	4.2	4.2	4.2
**Cystine (g)**	0.8	0.8	0.6
**Glutamic Acid (g)**	7.0	7.0	7.1
**Glycine (g)**	0.7	0.7	1.6
**Histidine (g)**	0.7	0.7	1.1
**Isoleucine (g)**	2.3	2.3	1.9
**Leucine (g)**	4.2	4.2	3.4
**Lysine (g)**	3.7	3.7	2.5
**Methionine (g)**	0.9	0.9	0.7
**Phenylalanine (g)**	1.2	1.3	2.3
**Proline (g)**	2.3	2.3	1.8
**Serine (g)**	2.0	2.0	2.0
**Threonine (g)**	2.7	2.7	1.5
**Tryptophan (g)**	0.7	0.7	0.4
**Tyrosine (g)**	1.2	1.2	1.5
**Valine (g)**	2.3	2.3	2.3

Supplemental Table S2. Muscle Function Raw Data.

Variable	Pre	Post (+0 h)	+24 h	+48 h	+72 h
**ISOMext (Nm)**					
**WI**	182.9 ± 15.4	152.9 ± 12.6	168.6 ± 15.4	175.0 ± 14.2	184.6 ± 18.0
**WH**	182.5 ± 10.3	164.3 ± 10.3	153.5 ± 12.6	173.7 ± 11.6	169.5 ± 14.6
**RP**	193.3 ± 15.4	157.7 ± 12.6	152.1 ± 15.4	144.3 ± 14.2	161.4 ± 18.0
**PL**	196.4 ± 15.4	144.8 ± 12.6	146.2 ± 15.4	148.5 ± 14.2	163.0 ± 18.0
**ISOMflex (Nm)**					
**WI**	86.2 ± 7.9	64.9 ± 8.9	66.7 ± 8.4	57.9 ± 9.1	60.6 ±8.7
**WH**	91.9 ± 6.5	68.7 ± 7.2	60.8 ± 6.9	64.0 ± 7.4	67.2 ± 7.1
**RP**	95.3 ± 7.9	69.1 ± 8.9	72.8 ± 8.4	51.4 ± 9.1	63.0 ± 8.7
**PL**	99.7 ± 7.9	73.8 ± 8.9	74.0 ± 8.4	70.9 ± 9.1	68.4 ± 8.7
**ISOK60ext (Nm)**					
**WI**	173.9 ± 10.9	140.8 ± 12.8	146.2 ± 14.0	152.1 ± 11.4	156.5 ± 13.8
**WH**	156.5 ± 8.9	142.1 ± 10.5	134.9 ± 11.4	134.2 ± 9.3	137.6 ± 11.3
**RP**	169.3 ± 10.9	136.8 ± 12.8	130.1 ± 14.0	108.6 ± 11.4	120.4 ± 13.9
**PL**	163.1 ± 10.9	132.0 ± 12.8	124.3 ± 14.0	120.6 ± 11.4	127.0 ± 13.9
**ISOK60flex (Nm)**					
**WI**	96.1 ± 8.0	71.1 ± 7.3	70.6 ± 8.4	61.5 ± 7.5	63.9 ± 8.5
**WH**	87.6 ± 6.5	72.8 ± 6.0	66.9 ± 6.9	60.1 ± 6.2	59.2 ± 7.0
**RP**	93.7 ± 8.0	62.6 ± 7.3	63.9 ± 8.4	43.5 ± 7.5	56.0 ± 8.5
**PL**	89.5 ± 8.0	70.0 ± 7.3	63.9 ± 8.4	56.0 ± 7.5	56.8 ± 8.5

Data are presented as mean ± SE.
